# The Androgen Receptor and VEGF: Mechanisms of Androgen-Regulated Angiogenesis in Prostate Cancer

**DOI:** 10.3390/cancers9040032

**Published:** 2017-04-10

**Authors:** Kurtis Eisermann, Gail Fraizer

**Affiliations:** 1 School of Biomedical Sciences, Kent State University, Kent, OH 44242, USA; keiserma@kent.edu; 2 Department of Biological Sciences, Kent State University, Kent, OH 44242, USA

**Keywords:** androgen receptor, AR, VEGF, angiogenesis, hypoxia, prostate cancer, CRPC

## Abstract

Prostate cancer progression is controlled by the androgen receptor and new blood vessel formation, or angiogenesis, which promotes metastatic prostate cancer growth. Angiogenesis is induced by elevated expression of vascular endothelial growth factor (VEGF). VEGF is regulated by many factors in the tumor microenvironment including lowered oxygen levels and elevated androgens. Here we review evidence delineating hormone mediated mechanisms of VEGF regulation, including novel interactions between the androgen receptor (AR), epigenetic and zinc-finger transcription factors, AR variants and the hypoxia factor, HIF-1. The relevance of describing the impact of both hormones and hypoxia on VEGF expression and angiogenesis is revealed in recent reports of clinical therapies targeting both VEGF and AR signaling pathways. A better understanding of the complexities of VEGF expression could lead to improved targeting and increased survival time for a subset of patients with metastatic castration-resistant prostate cancer.

## 1. Introduction

### Androgen Signaling and Angiogenesis

Hormones are known to regulate many genes involved in prostate cancer (PC) and prostate cancer progression to castration-resistant prostate cancer (CRPC). Classical androgen signaling requires the androgen receptor (AR) to bind to Dihydrotestosterone (DHT) or testosterone (T) and dissociate from heat shock proteins. AR is then phosphorylated and translocated to the nucleus where it binds DNA and other protein co-factors at dimeric AR recognition elements (ARE) and activates transcription of androgen responsive genes such as PSA, TMPRSS2, Nkx3.1, and FKBP5 [1,2,3,4,5,6]. Many co-factors that regulate AR signaling have been identified [7,8,9,10] including co-factors with chromatin remodeling functions such as histone acetyltransferases, methyltransferases, and demethylases recruited by the AR to regulate its signaling pathways. 

Identification of hormone-activated targets of the AR has been fueled by the need for useful markers of prostate cancer progression. While PSA remains the most widely used test for the presence of cancer of the prostate, it provides a large percentage of false positive results [11]. Thus, evidence of hormone responsive genes important in prostate cancer progression has been sought. One such androgen mediated gene is vascular endothelial growth factor (VEGF), a mitogen secreted by tumor cells that is essential for tumor angiogenesis and is necessary for tumor growth beyond 1–3 mm^3^ in volume [12]. Patients with metastatic prostate cancer have greater VEGF plasma levels than those with localized disease, as over-expression of VEGF contributes to tumor growth and metastasis [13]. VEGF is regulated by multiple transcription factors (TFs), that respond to changes in the micro-environment such as, HIF-1 (responsive to hypoxic conditions) [14], AR (responsive to hormone levels) [15,16,17], and other zinc-finger TFs that bind GC-rich promoter regions, e.g., Sp1 and WT1 [16,18]. This review will outline what is known about mechanisms of androgen regulation of VEGF and the importance of VEGF in angiogenesis in prostate cancer and prostate cancer progression. The relevance of delineating the androgen and VEGF pathways in PC is demonstrated in recent clinical trials targeting both AR and VEGF pathways (including HIF1-α) [19,20].

**Figure 1:**
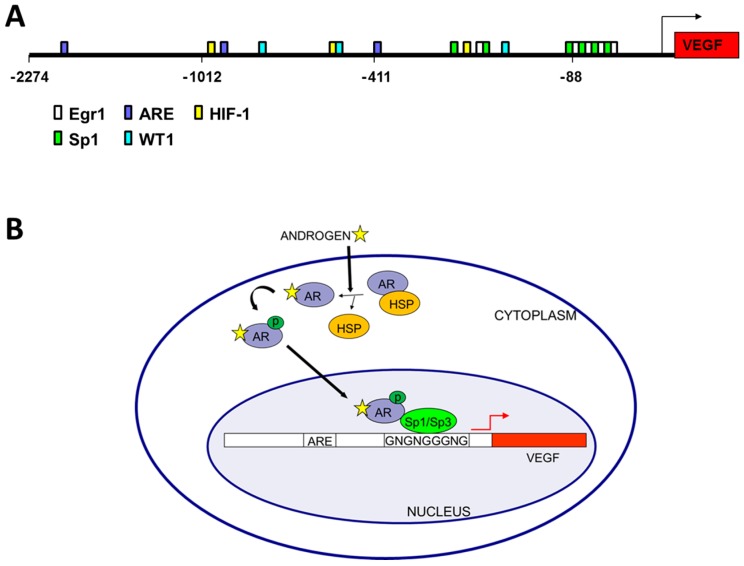
Androgen mediated regulation of vascular endothelial cell growth factor (VEGF) transcription. (**A**) Promoter analysis of VEGF. The VEGF promoter (VEGFA accession number AB021221) was downloaded from Ensembl and binding sites were predicted by MatInspector and located on the VEGF promoter sequence [50]. Potential androgen receptor binding sites (ARE), HIF1α binding sites (HIF-1) and zinc finger transcription factor binding sites (Sp1, Egr1, and WT1) thought to play a role in VEGF regulation are color coded according to the legend; (**B**) Model of androgen regulation of VEGF in prostate cancer showing the AR in a complex with Sp1 and bound to the GC-rich region of the VEGF core promoter. Note that ligand binding replaces HSP binding in the cytoplasm, but within the nucleus Sp1 binding recruits the AR to the core promoter region of the VEGF gene.

VEGF regulation is complex and occurs at both transcriptional and post-transcriptional levels [21,22,23]. While the VEGF promoter lacks a TATA-binding site, it contains a GC-rich core promoter region and additional distal enhancer sites including hypoxia response elements that bind HIF1-α [24] (Figure 1A). Transcriptional and post-transcriptional regulation of VEGF has been well studied and both genetic and epigenetic mechanisms have been identified. For nearly 20 years it has been known that androgen up-regulates VEGF expression [17,25,26]. However, the mechanism of activation, whether via classical or non-classical pathways, is not yet entirely understood. The VEGF promoter lacks canonical androgen receptor (AR) DNA binding sites (ARE) either dimeric inverted or direct repeats. Whether androgens may instead be activating VEGF through non-classical pathways via src/MAPK is also unclear [27]. However, VEGF is activated via multiple pathways both in normoxia and hypoxia conditions. Below we discuss the roles of epigenetic and transcription factors AR, Sp1 (specificity protein 1), WT1 (Wilms tumor gene 1) and HIF1-α Hypoxia inducible factor 1-α) in regulating VEGF expression in conjunction with hormone.

## 2. Androgen and Epigenetic Regulation of VEGF

### 2.1. VEGF Regulation by Histone Modifiers

AR co-factors either co-activate or co-repress AR target gene expression, and several of the AR co-factors do so by modifying histone proteins. One well studied epigenetic modifier of AR target gene expression is Lysine specific demethylase 1 (LSD1/KDM1A) which has been identified in complexes with ligand bound AR [28]. LSD1 demethylates repressive histone marks and thereby can increase AR dependent transcription [28,29]. However, since AR autoregulates its own expression, it is noteworthy that AR recruitment of LSD1 to the AR promoter itself leads to a negative feedback loop repression of AR transcription [30]. Thus, LSD1, like traditional transcription factors, acts to regulate transcription, but the repressive or enhancing consequences are gene promoter context specific. Nonetheless, LSD1 up-regulates VEGF-A expression in both hormone responsive PC cells such as LNCaP, or non-responsive PC3 cells [29].

Recently, protein arginine methyltransferase 5 (PRMT5) has been shown to activate AR expression and promote PC cell growth [31]. PRMT5 binds the proximal promoter of the AR gene in a complex with Sp1 and the chromatin remodeling enzyme Brg1. Since VEGF is transcriptionally activated by androgens, PRMT5 can be expected to indirectly up-regulate VEGF and angiogenesis as well. This would be consistent with elevated PRMT expression observed in PC compared to BPH, and suggestive of an oncogenic function [31]. Although epigenetic regulators of AR and VEGF have been identified, evidence of their direct interaction with the AR on the VEGF promoter has been limited to that described for LSD1 [29].

### 2.2. Post-Transcriptional Regulation of VEGF by mRNA Stabilizers

VEGF mRNA is typically short-lived with a half-life of 15–40 min [32], but VEGF mRNA message stability is enhanced by low oxygen levels (hypoxia) through the binding of stabilizing proteins to the 3’untranslated regions (3’UTR). Members of the ELAV family of RNA binding proteins, like HuR, and heterogeneous nuclear ribonucleoprotein L (hnRNPL) bind to the AU-rich elements of the 3’UTR [33,34]. One potential mechanism for VEGF mRNA stabilization by HuR binding is that these stabilizing proteins block binding by the de-stabilizing micro RNAs also known to bind the 3’UTR. Indeed the binding sites for HuR and miR-200b overlap and miR-200b can compete with HuR binding to suppress VEGF mRNA expression [35]. Similarly, competition for 3’UTR binding between the hnRNPL and the γ-IFN-activated inhibitor of translation complex (GAIT) has been referred to as a riboswitch [36]. Hypoxia elevates hnRNPL protein levels and they bind and stabilize VEGF mRNA which acquires a secondary structure that blocks binding by the repressive GAIT complex [36].

As an example of the complexity of VEGF regulation, the riboswitch region is also a binding area for several microRNAs that also compete with hnRNPL for binding at the VEGF 3’UTR [37]. Of note, this is not an AU-rich but rather a CA-rich region (CARE). Overall, multiple miRNAs that bind to the 3’UTR of VEGF have been identified (reviewed in [38]) but their sensitivity to androgen is not known. Interestingly, the AR primarily up-regulates mi-RNAs considered to be oncogenic (oncomirs) and but none of these have been reported to up-regulate VEGF. Recently, androgen has been shown to suppress a miRNA cluster (miR-99a/let7c/miR-125b2), but this suppression still enhances PC cell proliferation [39].

### 2.3. Translational Regulation of VEGF 

The relative importance of the 3’UTR region of VEGF for post-transcriptional regulation of VEGF is not greater than that of the 5’ UTR where two internal ribosome entry sites (IRES) permit cap independent translation of two separate translation start sites (AUG and upstream CUG sites) (reviewed in [38]). Of note, a sequence within the IRES-A promotes G-Quadruplex formation, conferring a suppressive structure on the VEGF 5’UTR [40]. Importantly, the 5’UTR is a critical regulatory area and in response to stress such as hypoxia, the IRES-B upstream of the CUG start sites will promote cap independent translation of the L-VEGF form encoding a longer isoform, that after proteolysis provides both an internal and the secreted VEGF peptide [41]. The clinical significance of the IRES-B was suggested when a single nucleotide polymorphism (SNP) was identified that suppressed the IRES-B function, reducing CUG translation initiation, and thereby decreasing L-VEGF protein levels. This SNP was associated with an elevated risk of prostate cancer [42].

Although this review will not cover the diversity of alternative VEGF isoforms, clearly the several alternative start codons, alternative splicing and the post translational proteolysis lead to a large number of variant VEGF protein isoforms with alternative functions (Reviewed in [38]). Use of the AUG translation initiation site is dependent upon specific exonic sequences that may be deleted in some alternatively spliced transcripts. For example, the alternatively spliced transcript encoding VEGF 121 (the diffusible form of VEGF lacking exons 6 and 7 encoding the heparin binding domains) cannot be translated from the AUG initiation site, but rather its translation initiates from an upstream CUG site [43]. The wide variety of VEGF isoforms have a variety of functions differentially affecting angiogenesis, varying from distal activity (EGF 121), to locally restricted activity (VEGF 189), to antiangiogenic activity (VEGF 165b). The role of androgen in altering VEGF isoform ratios is not yet understood but can be expected to be of clinical significance.

## 3. Transcription Factors that Regulate Androgen Induction of VEGF Expression

### 3.1. Sp1 

Androgen treatment of prostatic fibroblasts and LNCaP cells significantly increases VEGF mRNA expression levels [15,16,44,45]. Additionally, VEGF protein levels have been demonstrated to be up-regulated after treatment of LNCaP cells with hormone [17], and the androgen antagonist flutamide blocks this up-regulation [46]. The mechanism of androgen-mediated regulation of VEGF expression, however, is less well understood. Three potential monomeric ARE half-sites were predicted by in silico analyses within the VEGF promoter, (Figure 1A) similar to sites reported in other gene promoters [47,48,49]. Furthermore, the androgen analog R1881 was shown to up-regulate both the proximal and distal VEGF promoter activity in 22Rv1 and LNCaP cells [18,24]. Taken together these results indicate the VEGF promoter is hormone responsive. 

Interestingly, regions in the VEGF promoter near predicted ARE half-sites contain G-rich binding sites for other zinc finger transcription factors (ZFTF) such as Sp1, EGR1 (early growth response 1) or WT1 that could potentially interact with the AR. Non-classical AR half-sites were also identified adjacent to G-rich WT1/EGR1/Sp1 sites in 8 of 11 promoters analyzed, including VEGF [50]. Co-transfection of WT1 expression plasmids enhances VEGF promoter activity [18,24], with addition of the androgen analog R1881 increasing WT1 effectiveness, and mutation of a WT1 site reducing VEGF promoter activity [18]. These results were consistent with chromatin immunoprecipitation of WT1, Sp1 and AR at the VEGF promoter and co-immunoprecipitation of AR with Sp1 or WT1 [18,50].

Surprisingly, the ARE half-sites identified in the VEGF promoter are not required for hormone induction of VEGF expression, as site directed mutagenesis failed to eliminate hormone response [16]. Rather a single GC-box in the core promoter is essential for hormone responsiveness of the VEGF promoter [16]. This indicates that the AR is not bound to an ARE binding site, but rather is tethered via a ZFTF, which is bound to the GC boxes (Sp1/Sp3 binding sites) (Figure 1B). This GC-rich VEGF core promoter lacking ARE half-sites is responsive to androgen stimulation of PC cells, inhibited by the anti-androgen casodex [16], and is also the region of estrogen responsiveness in breast cancer cells [51,52]. In addition to lacking canonical dimeric ARE sites, the VEGF promoter also lacks canonical estrogen receptor (ER) binding sites [51,52]. Similarly, VEGF regulation by estrogen in endometrial and breast cancer cells involves interactions of ER-α and Sp1 (or Sp3) with GC boxes in the core promoter region of VEGF [51,52]. VEGF mRNA levels are significantly induced in ZR-75 breast cancer cells treated with estradiol, and the intact GC-rich core VEGF promoter region is required for such activation. The relevance of Sp1 and Sp3 in estradiol regulation of VEGF in breast cancer was demonstrated by binding assays in vitro (by EMSA) and in vivo (by ChIP) [51,52]. The VEGF core promoter contains four Sp1 binding sites and mutation of only the Sp1 site closest to the transcription start site inhibited androgen activation of VEGF in PC cells, while other adjacent sites were not required for hormone response [16]. Together, these results indicate a mechanism of androgen-mediated induction of VEGF expression in PC cells involving interaction of the AR with a specific, critical Sp1 binding site in the VEGF core promoter region [16] (Figure 1B).

### 3.2. Hypoxia (HIF-1α)

VEGF expression is up-regulated in response to hypoxia and this is mediated by the stabilization of the transcription factor hypoxia-inducible factor 1 (HIF-1α) that up-regulates transcription of VEGF via binding at HIF-Responsive elements. Importantly, HIF-1α itself is up-regulated by DHT both via transcript stabilization [53] and via an autocrine loop involving EGF-R and AKT [46]. The clinical importance of HIF-1α expression in prostate cancer has been demonstrated and HIF-1α has been examined as a potential prognostic marker, being elevated in high grade PIN and not BPH [54]. Response to androgen deprivation therapy in mice with CWR22RV1 xenografts, suggests that AR may regulate HIF-1α levels, as expression of both AR and HIF-1α target genes were affected even outside of hypoxic tumor areas [55].

Conversely, substantial evidence exists for the effect of HIF-1α (and hypoxia) on AR signaling. Combined hypoxia and hormone treatment synergistically increased PSA levels [56]. Hypoxia increases transcriptional activity of ARE-luciferase reporters in low or high DHT conditions, but has no effect in the absence of DHT [57]. Thus, androgen signaling is influenced by hypoxia, which itself up-regulates VEGF expression. Overall, this suggests that VEGF response to hypoxia may be mediated in part by HIF-1α but in the case of endocrine tumors, also by hormone effects on HIF-1α [53].

## 4. AR Variants

Currently, a family of AR splice variants are being identified that lack the LBD, but arise in patients undergoing androgen deprivation therapy [58]. These splice forms lacking the LBD region have been seen in BPH and localized prostate cancers, but are up-regulated in castration resistant prostate cancer [59,60,61]. The presence of these variants is significant, as patients with a high level of AR-V7 and ARv567es expression have a shorter survival expectancy than CRPC patients lacking these AR variants [59]. Additionally, AR-V7 (the AR-V most commonly expressed in clinical specimens) has been shown to be involved in resistance to both enzalutamide and abiraterone in clinical studies [62,63]. Importantly in CWR22Rv1 cells, which contain AR splice variants (including AR-V7) [61,64,65], we have shown that Sp1 and the AR interact to activate the VEGF promoter [16]. If AR variants interact with Sp1 (either directly or through complex formation with full-length AR) they could influence VEGF expression in response to hormone. Additionally, these AR variants can recruit and form complexes with co-factors that have chromatin remodeling functions discussed above, such as histone acetyltransferases, methyltransferases, and demethylases, potentially impacting the epigenetic regulation of the AR and VEGF [29]. Thus, it will be important to determine if these novel splice variants of the AR are involved in VEGF regulation in CRPC, particularly if improved response is observed in clinical trials with CRPC patients being treated with both anti-androgens and VEGF inhibitors. 

## 5. Relevance of Dual Targeting of Hormone Signaling and VEGF in PC Tumor Angiogenesis

Metastatic PC is associated with higher VEGF levels than localized disease [66,67,68]. Thus, anti-VEGF therapies have been the target of multiple clinical trials for treatment of men with CRPC. Bevacizumab is a monoclonal antibody to VEGF-A which has been shown to decrease tumor volume in many cancers. However, in clinical trials for treatment of CRPC, it has not improved the overall survival time of patients getting chemotherapy (docetaxol) along with the immunosuppressant prednisone [69]. Therefore, it is thought that angiogenesis may play a smaller role in CRPC than other cancers and current studies are investigating dual targeting of both androgen signaling and VEGF.

**Figure 2:**
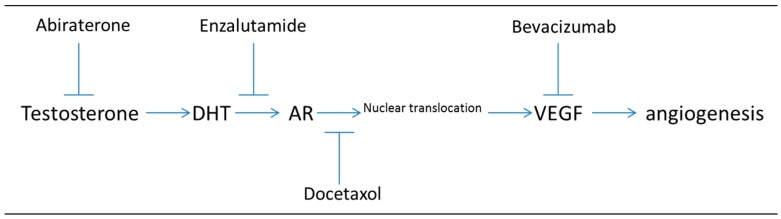
Targeting both VEGF induction of angiogenesis and androgen synthesis or AR signaling inhibits two critical signaling pathways in prostate cancer (PC) progression. Note that hypoxia induced VEGF can also be suppressed by targeting HIF1α with HIF1 inhibitors (not shown).

Studies targeting both the androgen signaling pathway (with bicalutamide or enzalutamide) and VEGF (either directly with a VEGF inhibitor or indirectly through HIF1-α inhibition) have recently been performed [19,20]. Figure 2 illustrates the steps at which dual drug targeting could impact both (1) the androgen signaling pathway (through abiraterone blocking androgen synthesis, enzalutamide binding to AR, or docetaxel inhibiting microtubule driven transport of AR-androgen complex) and (2) the angiogenic pathway (through bevacizumab blocking VEGF binding). The effects of targeting both the AR signaling pathway and HIF-1α pathway have also been investigated and the authors found that combinatorial targeting both of these pathways lead to greater inhibition of prostate cancer cell growth than either one alone in both LNCaP and CWR22Rv1 cells [20]. Also, this study determined that VEGF protein levels were significantly reduced in the presence of both enzalutamide and siHIF-1α, suggesting that VEGF could be a biomarker for enzalutamide response [20]. 

A new phase II clinical trial of patients with recurring prostate cancer treated with or without the VEGF inhibitor Bevacizumab after ADT revealed that ADT combined with Bevacizumab resulted in an increased relapse free survival rate, although modestly, compared to ADT alone [19]. These results suggested that combining ADT with Bevacizumab could prolong the off-ADT-cycle during intermittent ADT and thus benefit a subset of patients that have hormone sensitive prostate cancer. These studies demonstrate the need for understanding mechanistically the relationship between AR and VEGF and how they interact in CRPC patients.

## 6. Conclusions

Treatment of CRPC involves targeting many factors and signaling pathways which are still being uncovered, but dual targeting of both AR and VEGF signaling should result in better efficacy for patients than either one alone. Mechanistically, it appears that androgen induction of VEGF is regulated through AR complex formation with Sp1 in the core promoter region in prostate cancer cells and not via ARE binding sites in the distal VEGF promoter. Therefore, addition of Sp1 or HIF-1α inhibitors could further add to the significant effect seen by targeting AR signaling with enzalutamide and VEGF with Bevacizumab. Further delineation of the mechanism(s) involved in the progression of CRPC and the pathways utilized will help to produce even better treatment plans for this subset of patients.
